# Wien effect of Cd/Zn on soil clay fraction and their interaction

**DOI:** 10.1186/s12932-018-0050-y

**Published:** 2018-02-13

**Authors:** Tingting Fan, Chengbao Li, Juan Gao, Dongmei Zhou, Marcelo Eduardo Alves, Yujun Wang

**Affiliations:** 1 Key Laboratory of Soil Environment and Pollution Remediation, Institute of Soil Science, Chinese Academy of Sciences, Nanjing, 210008 China; 2 University of Chinese Academy of Sciences, Beijing, 100049 China; 3 Nanjing Institute of Environmental Science, State Environmental Protection Administration, Nanjing, 210042 China; 4 Department of Exact Sciences, ‘Luiz de Queiroz’ College of Agriculture–ESALQ/USP, Piracicaba, SP 13418-900 Brazil

**Keywords:** Wien effect, Zn^2+^, Cd^2+^, Combined pollution, Soil particles

## Abstract

**Background:**

The coexistence of Cd^2+^ and Zn^2+^ ions in nature has a significant influence on their environmental behaviors in soils and bioavailability for plants. While many studies have been done on the mutual toxicity of Cd^2+^ and Zn^2+^, few studies can be found in the literature focused on the interaction of Cd^2+^ and Zn^2+^ on soil clay fractions especially in terms of energy relationship.

**Results:**

The binding energies of Cd^2+^ on boggy soil (Histosols) particles and Zn^2+^ on yellow brown soil (Haplic Luvisols) particles were the highest, while those of Cd^2+^ and Zn^2+^ on paddy soil (Inceptisols) particles were the lowest. These results indicated that Cd^2+^ and Zn^2+^ have a strong capacity to adsorb in the solid phase at the soil–water interface of boggy soil and yellow brown soil, respectively. However, both Cd^2+^ and Zn^2+^ adsorbed on paddy soil particles easily release into the solution of the soil suspension. Unlike the binding energy, the higher adsorption energies of ions in boggy and yellow brown soils showed a weak binding force of ions in boggy soil and yellow brown soil. A 1:1 ratio of Cd^2+^ to Zn^2+^ promotes the mutual inhibition of their retentions. Cd^2+^ and Zn^2+^ have high mobility and bioavailability in paddy soil and yellow drab soil (Ustalfs), whereas they have high potential mobility and bioavailability in boggy soil and yellow brown soil.

**Conclusion:**

In the combined system, Zn^2+^ had preferential adsorption than Cd^2+^ on soil clay fractions. Boggy soil and yellow brown soil have a low environmental risk with lower mobility and bioavailability of Cd^2+^ and Zn^2+^ while paddy soil and yellow drab soil present a substantial environmental risk. In the combined system, Cd^2+^ and Zn^2+^ restrain each other, resulting in the weaker binding force between ions and soil particles at a 1:1 ratio of Cd^2+^–Zn^2+^.

## Background

In China, about 13% of soils included in the national assessment of soil contamination hold excessive amounts of inorganic pollutants [1]. Most of these contaminants are metals and metalloids that can be found in fertilizers [2] or in several classes of hazardous residues that are improperly disposed of in the soils [3]. Such elements can pose a threat to human health because of their accumulation throughout the food chain [4–7].

Both cooperative and antagonistic toxic effects are reported for soil pollutants such as those for Cd^2+^ and Zn^2+^. Turner [8] found that Cd^2+^ might increase Zn^2+^ uptake in some plants as a result of root damage in the higher Cd^2+^. Wu et al. [9] suggested that Cd^2+^ enhances Zn^2+^ accumulation in chloroplast (FII), whereas Zn^2+^ addition decreases the concentration of Cd^2+^ in root trophoplast (FII). Van Gestel and Hensbergen [10] observed that Cd^2+^ and Zn^2+^ had antagonistic and synergistic effects on the growth and reproduction of the Collembola, respectively.

The adsorption process at the solid–water interface strongly affects both mobility and bioavailability of pollutants in soil. Despite this, most research efforts in this field using empirical sorption isotherms or surface complexation models have focused on the behavior of single adsorbate [11–14]. Even the direct assessment of binding strengths between adsorbates and soil particles does not account for the competitive effects that take place in multi-adsorbate environments such as those found in soils [15–19].

Sorption studies carried out in multi-reactive media can better characterize the behavior of pollutants in soils. The use of soil samples and multi-metal solutions allowed Gomes et al. [20] to assess not only the competition effects on the metal distribution coefficients but also the effects of intrinsic cation properties such as the trend to hydrolysis and the softness parameter of Misono on the preferential metal adsorption. Using a competitive two-metal adsorption isotherm Ming et al. [21] observed that the co-adsorption of Zn^2+^ enhanced the Cd^2+^ mobility in soils.

The measurement of the Wien effect in colloidal suspensions is a straightforward and rapid method established by Li et al. [22] for determining binding energies associated with ion adsorption. This approach is based on the so-called Wien effect and colloidal electrolyte theory as proposed by Marshall and Krinbill [23]. The Wien effect is a deviation from Ohm’s law characterized by the enhancement of the electrical conductivity (EC) of a suspension in response to the application of an increasing electrical field (E) [24]. We have been successfully measuring this effect in suspensions of whole soils and soil minerals during the last decade [18, 25].

The Wien effect curves (EC vs. E) allows for quantifying the mean free ion binding and adsorption energies and the intensity of ion stripping from soil particles [15, 18, 22, 26, 27]. However, so far, this procedure was only applied to single metal systems. Therefore, in this paper we used the Wien effect to evaluate the co-adsorption impacts on the binding energies of Cd^2+^ and Zn^2+^ retained on soil clay fractions.

## Materials and methods

### Soils

Surface soil samples (0–0.2 m) were collected in four locations of Jiangsu Province, China, air-dried, ground, and passed through a 60-mesh sieve. Both soil pH and electrical conductivity (EC) values were measured in aqueous suspensions at the soil/water ratio of 1:2.5. Free iron was extracted with dithionite-citrate-bicarbonate solution whereas soil organic matter (SOM) was quantified through oxidation with potassium dichromate and sulphuric acid at 170–180 °C [28, 29]. The ethylenediaminetetraacetic acid (EDTA)-ammonium acetate exchange method was used to measure the soil cation exchange capacity (CEC) [29]. Dissolved organic carbon (DOC) was extracted at a soil/water ratio of 1:10 and determined with a Vario Cube TOC analyzer (Elementar Inc.). The results of the above evaluations are summarized in Table 1. The sand-sized (ϕ > 0.050 mm) particles were separated through wet sieving and then the clay-sized ones (ϕ < 0.002 mm) were isolated from the silt-sized particles (0.002 mm ≤ ϕ ≤ 0.050 mm) through sedimentation [30]. The minerals present in the clay fraction were identified by X-ray diffraction (Table 2).

**Table 1:** Properties of the studied soil samples

Location	Chinese soil type	US soil type	Depth (cm)	EC (μs cm^−1^)	pH	CEC (cmol kg^−1^)	OM (%)	DOC (mg L^−1^)	Fe_DCB_ (g kg^−1^)	Clay (%)
Liyang	Paddy soil	Inceptisols	0–20	233.27	3.36	15.8	2.40	248	7.97	N
Jianhu	Boggy soil	Histosols	0–20	495.77	6.22	26.4	2.98	149.5	6.77	13.1
Xuyi	Yellow drab soil	Ustalfs	0–20	147.41	5.72	17.8	2.17	251.5	7.91	10.7
Nanjing	Yellow brown soil	Haplic Luvisols	0–20	51.07	5.77	16.2	1.08	197	8.68	13

**Table 2:** Mineralogical composition of the clay fractions of the studied soils

Soil	Montmorillonite	Vermiculite	Hydromica	Kaolinite	Chlorite	Quartz	Feldspar
	%						
Paddy soil	4	4	18	31	31	12	/
Boggy soil	20	6	24	11	26	13	/
Yellow brown soil	0	12	25	19	29	14	1
Yellow drab soil	7	5	22	24	30	11	1

**Fig. 1:**
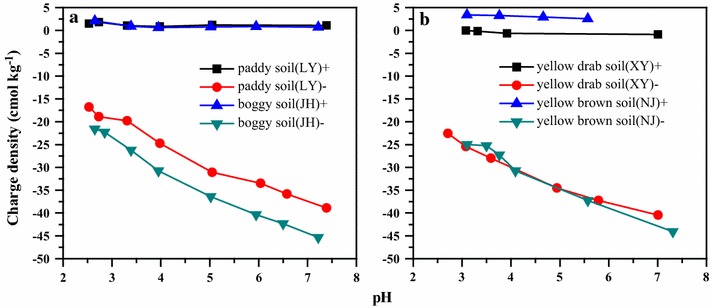
Negative and positive charge densities of clay fractions (< 2 μm) of the studied soils (**a**) paddy soil (Inceptisols) and boggy soil (Histosols) (**b**) yellow drab soil (Ustalfs) and yellow brown soil (Haplic Luvisols) as a function of pH

Before the saturation experiments, the clay particles were washed with 0.001 M HNO_3_ and 0.0001 M HNO_3_ to remove adsorbed cations and then their charge densities were determined using the modified Schofield method [31]. Whereas the negative charges were enhanced on the particles with the increase of suspension pH, the insignificant amounts of positive charges kept almost constant (Fig. 1). Such irrelevance of positive surface charges is a prerequisite for the Wien effect measurements.

### The preparation of soil clay fractions saturated with Cd^2+^ and Zn^2+^ and suspensions

The soil clay fractions were suspended in solutions containing 0.4 mol L^−1^ of positive charges from both Cd^2+^ and Zn^2+^. The solutions were prepared through the dissolution of amounts of reagent grade Cd(NO_3_)_2_^·^4H_2_O and Zn(NO_3_)_2_^·^6H_2_O sufficient for obtaining Cd^2+^:Zn^2+^ molar ratios of 3:1 [0.15 mol L^−1^ Cd(NO_3_)_2_ and 0.05 mol L^−1^ Zn(NO_3_)_2_], 1:1 Cd:Zn [0.10 mol L^−1^ Cd(NO_3_)_2_ and 0.10 mol L^−1^ Zn(NO_3_)_2_], and 1:3 Cd:Zn [0.05 mol L^−1^ Cd(NO_3_)_2_ and 0.15 mol L^−1^ Zn(NO_3_)_2_]. The surfaces of the clay fraction particles were saturated with Cd^2+^ and Zn^2+^ through three successive batches that included agitation, centrifugation, supernatant disposal and resuspension in the initial solution. Thereafter, the non-adsorbed ions were removed from particle surfaces through successive batches with deionized water that were repeated until the EC stabilization of the supernatants in a minimal value. The saturated particles were then oven-dried, ground, sieved and analyzed for their total amounts of Cd and Zn in an atomic absorption spectrophotometer Hitachi 2000 after microwave-assisted acid dissolution [32]. Aqueous suspensions (10 g L^−1^) of the saturated particles were prepared in 50-mL centrifuge tubes, agitated for 30 min and then sonicated for 45 min. After 10 additional 1-h agitations intercalated with 23 h of standing, the equilibrated suspensions were used in the measurements of the Wien effect for assessing binding energies of Cd and Zn cations adsorbed to the soil clay fractions.

### Wien effect measurements

The electrical conductivity under strong electrical fields was measured with the SHP-2 (short high-voltage pulse) apparatus. Both the SHP-2 apparatus features and the measuring procedure were detailed elsewhere [15, 19, 22, 27, 33]. Before measuring the Wien effect with the apparatus, the weak-field EC of the sample was determined with a regular conductivity meter (DDS-310, Shanghai Kangyi instrument Co.). The measurements were conducted at 25 °C to ensure that the suspension resistance was within the range from 200 Ω to 20 kΩ. The strong-field ECs measurements were carried at 25 °C by applying a voltage drop that increased from 1.0 kV up to the sparking occurrence (dielectric breakdown). The electrode spacing was kept constant at 1 mm. The first set of measurements of the Wien effect was carried out from low to high field strengths whereas the second one was performed in reverse order to eliminate possible effects of long-term heating and other irreversible phenomena. In consideration of the lower energy contributed by the applied electrical field, the outer-sphere complexes were examined by the Wien effect method. After the measurements of the Wien effect the suspensions were centrifuged and the supernatants were filtered and analyzed for Cd and Zn. All measurements were performed in duplicate.

### Binding energies of adsorbed Cd^2+^ and Zn^2+^

The direct relationship between ion activity and electrical conductivity was considered for the evaluation of both mean Gibbs free binding and adsorption energies. The evaluation of the mean Gibbs free binding energies in samples saturated with two metals is based on the assumption that the cation contribution to EC_0_ is proportional to its concentration in the equilibrium solution. Then, the following equations can be considered:

1 $${\text{EC}}_{0i} \, = \,{\text{C}}_{i} \lambda_{i} /\left( {{\text{C}}_{i} \lambda_{i} \, + \,{\text{C}}_{j} \cdot \lambda_{j} } \right)\, \times \,{\text{EC}}_{0}$$

2 $${\text{EC}}_{0j} \, = \,{\text{EC}}_{0} \,{-}\,{\text{EC}}_{0i}$$

where, EC_0*i*_ and EC_0*j*_ are the respective weak-field electrical conductivities caused by cations *i* and *j* (mS cm^−1^); C_*i*_ and C_*j*_ are the respective concentrations of cations *i* and *j* in the equilibrium solution (mol L^−1^); λ_*i*_ and λ_*j*_ are the respective equivalent conductivities of cations *i* and *j* (mS L cm^−1^ mol^−1^); and EC_0_ is the weak-field electrical conductivity associated with the Wien effect (mS cm^−1^).

The mean Gibbs free binding energy of each cation (ΔG_*bi*_) is calculated with the following equation:

3 $$\Delta {\text{G}}_{bi} \, = \,{\text{RTln}}\left( { 2 {\text{M}}_{i} {\text{D}}_{\text{p}} \lambda_{i} } \right)/{\text{EC}}_{0i}$$

where, R is the universal gas constant (8.315 J mol^−1^ K^−1^); T is the temperature (K); M_*i*_ is the adsorbed amount of cation *i* (mol kg^−1^); D_p_ is the suspension particle density (g L^−1^); λ_*i*_ is the equivalent conductivity of the cation *i* (mS L cm^−1^ mol^−1^); and EC_0*i*_ is the weak-field electrical conductivity caused by cation *i*.

For the sake of clarity, the minus sign of Eq. (3) was omitted and to the binding/adsorption energies will be assigned positive signs. The binding energy reflects the distribution proportion of metal ions on the interface of solid–solution. In Eq. (3), the numerator represents the EC expected from the contribution of all metal ions in the suspension under ideal condition. The denominator represents the EC contributed by the dissolved metal ions. The larger the binding energy is, the smaller the denominator is, that is, there are less dissolved metal ions in suspensions.

### Adsorption energies of adsorbed Cd^2+^ and Zn^2+^

Given the non-selectivity of the electrical conductivity measurement, in a mixed system only the total adsorption energy of cations can be assessed, similar to the adsorption energy of single ions.

If one defines states (1) and (2) of the suspension as the electrical conductivities under the weak and the strong electrical fields, respectively, the mean Gibbs free adsorption energy can be evaluated from:

4 $$\Delta {\text{G}}_{\text{ad}} \, = \,{\text{RTln}}\left( {{\text{EC}}/{\text{EC}}_{0} } \right)$$

where, EC and EC_0_ are the respective electrical conductivities of the suspension under the strong and the weak electrical fields.

Equation (4) enables one to evaluate the mean free adsorption energy of all cations stripped off from the soil clay fraction particles as the electrical field increased from zero to E. The application of Eq. (4) to a series of measurements of the Wien effect, EC(E), can provide a spectrum of the cation adsorption energies, ∆Gads(E). If the metal strongly adsorbs to the soil clay fraction particles, fewer ions will be dissolved at a weak E and the EC enhancement at the high E will be substantial, i.e. the adsorption energy will be high.

## Results and discussion

### Cd^2+^ and Zn^2+^ on the soil clay fraction particles and in the equilibrium solutions

The amounts of Cd^2+^ and Zn^2+^ on clay particles and in equilibrium solutions as well as their distribution coefficients are shown in Table 3. The combined amounts of Cd^2+^ and Zn^2+^ on the clay-sized particles ranged from 122.1 to 173.3 mmol kg^−1^ and conformed to the CEC values determined for the whole soil samples. The Cd^2+^ to Zn^2+^ ratios in the clay-sized particles also agreed with the expected ones. The total cation concentrations in equilibrium solutions ranged from 0.008 to 0.170 mmol L^−1^.

**Table 3:** The contents of Cd^2+^ and Zn^2+^ in soil colloid particles and corresponding supernatant, the binding energies of Cd^2+^ and Zn^2+^ on different soil colloid particles at various ratio of Cd^2+^ and Zn^2+^, related parameters to calculate the binding energy

Cd^2+^/Zn^2+^	Soil type	EC_0_ (mS cm^−1^)	Metal in colloid particles (mmol kg^−1^)		EC_iu_ (mS cm^−1^)		Metal in supernatant (mmol L^−1^)		EC_i0_ (mS cm^−1^)		△G_bi_ (kJ mol^−1^)	
			Cd^2+^	Zn^2+^	Cd^2+^	Zn^2+^	Cd^2+^	Zn^2+^	Cd^2+^	Zn^2+^	Cd^2+^	Zn^2+^
1:1	Boggy soil	0.0088	75.24	79.17	0.0813	0.0836	0.0126	0.0175	0.0037	0.0051	7.64	6.95
	Paddy soil	0.0129	60.21	61.84	0.0650	0.0653	0.0445	0.0444	0.0065	0.0064	5.70	5.77
	Yellow brown soil	0.0079	62.68	64.03	0.0677	0.0676	0.0124	0.0117	0.0041	0.0038	6.95	7.15
	Yellow drab soil	0.0122	66.45	69.14	0.0718	0.0730	0.0835	0.0861	0.0061	0.0061	6.12	6.15
1:3	Boggy soil	0.0125	42.91	127.56	0.0463	0.1347	0.0013	0.0064	0.0021	0.0104	7.66	6.35
	Paddy soil	0.0128	33.74	92.28	0.0364	0.0974	0.0055	0.0182	0.0030	0.0098	6.17	5.70
	Yellow brown soil	0.0111	37.51	107.95	0.0405	0.1140	0.0076	0.0241	0.0027	0.0084	6.72	6.47
	Yellow drab soil	0.0141	39.37	113.31	0.0425	0.1197	0.0020	0.0086	0.0028	0.0113	6.78	5.84
3:1	Boggy soil	0.0114	127.64	45.69	0.1379	0.0482	0.0074	0.0024	0.0087	0.0027	6.86	7.14
	Paddy soil	0.0147	100.78	32.89	0.1088	0.0347	0.0148	0.0046	0.0113	0.0034	5.62	5.76
	Yellow brown soil	0.0153	112.25	37.92	0.1212	0.0400	0.0091	0.0025	0.0121	0.0032	5.72	6.23
	Yellow drab soil	0.0156	115.42	36.19	0.1247	0.0382	0.0123	0.0033	0.0124	0.0032	5.73	6.12

### Electrical conductivity of suspensions as a function of field strength

The weak-field electrical conductivity (EC_0_) is proportional to the concentration of dissolved ions found in solution. Most of EC_0_ values of the suspensions containing clay-sized particles extracted from the paddy soil (Inceptisols) and yellow drab soils (Ustalfs) were the highest measured ones (Table 3). This finding could be due to the fact that soils had substantial amounts of dissolved organic matter which, in turn, carries electrical charges and forms soluble complexes with metals that are liable to ionize [16].

**Fig. 2:**
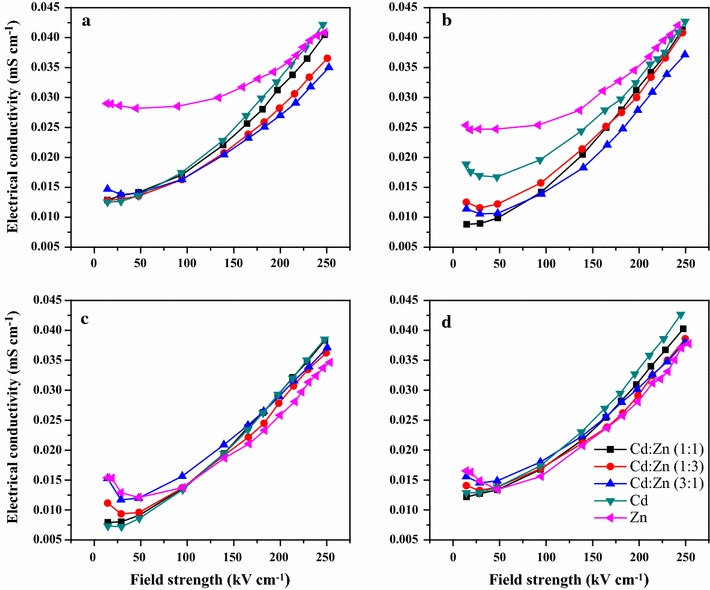
Dependence on field strength of electrical conductivity of suspension (10 g L^−1^) of clay fractions (< 2 μm) of paddy soil (Inceptisols) (LY) (**a**), boggy soil (Histosols) (JH) (**b**), yellow brown soil (Haplic Luvisols) (NJ) (**c**) and yellow drab soil (Ustalfs) (XY) (**d**) saturated with different ratios of Cd^2+^–Zn^2+^ in deionized water

The ECs of all studied suspensions were almost even under lower field strength values and began to increase from threshold ones (Fig. 2). The EC increase rate was small under weak field and became high when E turned strong. Such behavior is ascribed to the coexistence of a small number of loose ions with a substantial amount of strongly bound ones at the solid–liquid interface, which agrees with the ion distribution in the electrical double layer of soil clay fractions [34].

### Mean Gibbs free binding energy

**Fig. 3:**
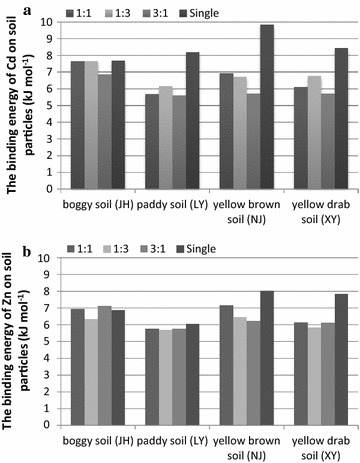
The binding energies of Cd^2+^ (**a**) and Zn^2+^ (**b**) on colloid particles of different soil types in single and combined systems

In all soils, the binding energies of Cd^2+^ in the absence of Zn^2+^ ranged from 8.2 to 9.9 kJ mol^−1^ and were greater than those found for Zn^2+^ in the absence of Cd^2+^ (6.1–8.0 kJ mol^−1^) (Fig. 3), which was consistent with the previous results reported by Fan et al. [16, 17]. On the other hand, as shown in Table 3, for all soils and Cd^2+^:Zn^2+^ ratios, the binding energies of Cd^2+^ ranged from 5.6 to 7.7 kJ mol^−1^ being the largest one related to the boggy soil (Histosols) and the smallest one verified in the paddy soil. Likewise, the binding energies of Zn^2+^ ranged from 5.7 to 7.2 kJ mol^−1^, with the largest one for the yellow brown soil (Haplic Luvisols) and smallest one for the paddy soil. As Table 2 shown, both yellow brown soil and boggy soil have high content of montmorillonite–vermiculite group mineral with more charge and large expansibility, while paddy soil has high content of kaolinite group mineral with less charge. Therefore, more metal ions adsorbed on yellow brown soil or boggy soil than paddy soil.

The binding energies of Cd^2+^ and Zn^2+^ in the mono-metal systems were higher than those in the competitive systems. Shaheen et al. [35, 36] observed lesser retentions of Cd^2+^ and Zn^2+^ in the competitive systems. Furthermore, the Cd^2+^ binding energy was lower in the competitive system while that of Zn^2+^ had no significant reduction compared to that observed in the single system. Antoniadis and Tsadilas [37] also obtained similar results, mainly because the binding strength between Cd^2+^ and soil particles was smaller and prone to be affected by competitive ions.

Boggy soil and yellow brown soil showed higher affinities for Cd^2+^ and Zn^2+^, respectively, while paddy soil has lower affinity for both cations. In other words, boggy soil and yellow brown soil attracted Cd^2+^ and Zn^2+^, respectively. On the other hand, both Cd^2+^ and Zn^2+^ tend to escape from the particles of paddy soil into soil solution.

**Fig. 4:**
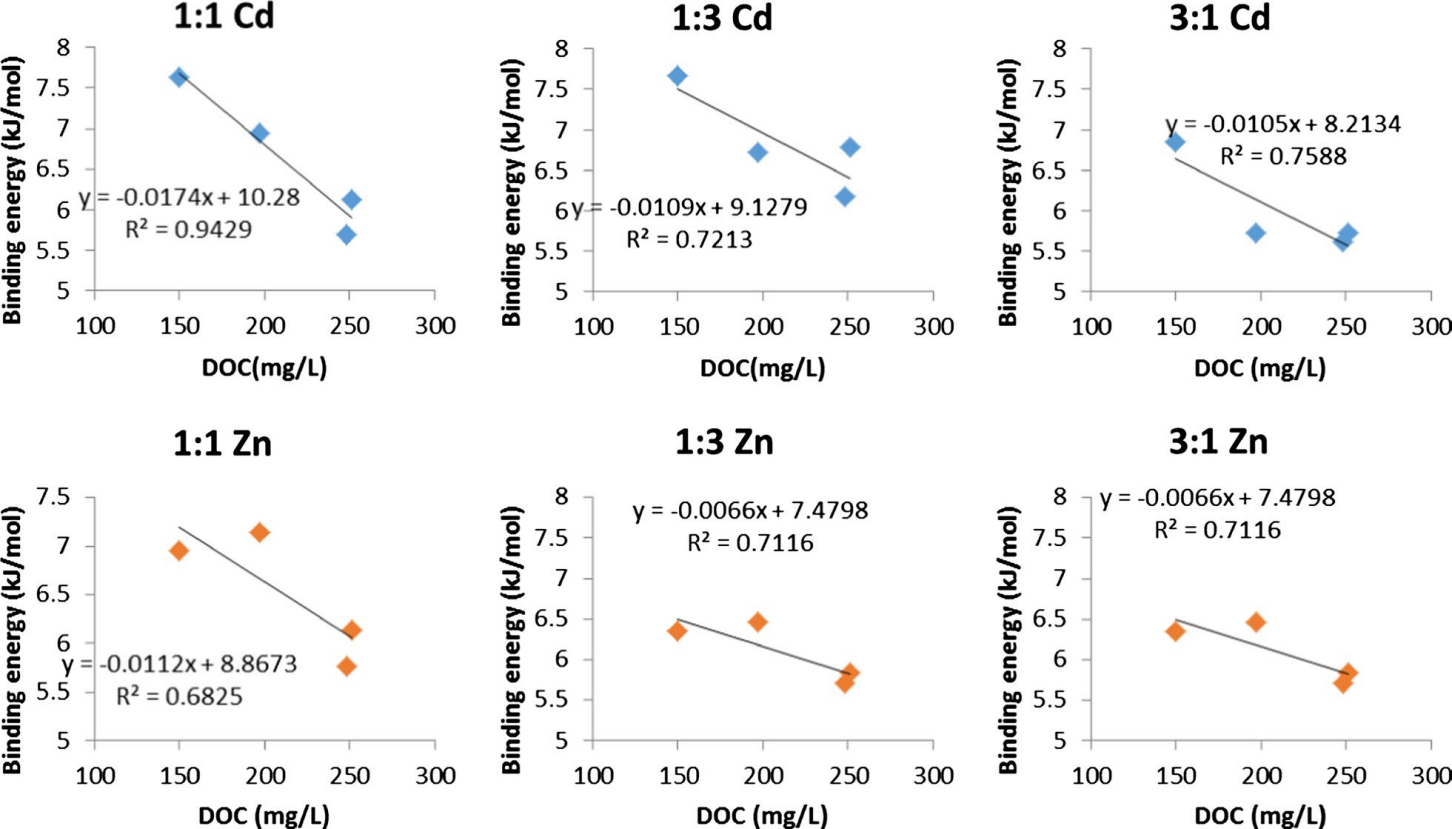
The linear relationship between the binding energy of Cd^2+^ or Zn^2+^ in the combined system with different Cd:Zn molar ratio and the DOC content in soil clay fractions

Soil is a multi-component system comprised of clay minerals, organic matter and Fe/Al oxides. These components have different selectivity towards Cd^2+^ and Zn^2+^. Thereinto, soil organic matter has higher affinity for Cd^2+^ at lower pH, while clay minerals and Fe/Al oxides have stronger affinities for Zn^2+^ than Cd^2+^ at higher pH and lower surface coverage [38]. The pHs of suspensions prepared with soil clay fractions saturated with Cd^2+^ and Zn^2+^ were 4.49 and 5.31. Therefore, soil organic matter may be the crucial factor affecting the binding energies of Cd^2+^ and Zn^2+^ on various soil clay fractions. As Table 1 shows, the organic matter content of boggy soil, especially of the non-dissolved one, is larger than that in yellow brown soil. Moreover, the correlation between the binding energy of Cd^2+^ or Zn^2+^ in the combined system with different Cd:Zn molar ratios and the DOC contents in soil clay fractions was analyzed and shown in Fig. 4. The negative linear relationship was found, which means that the higher content of DOC is, the smaller the binding energy of Cd^2+^ or Zn^2+^ is. The presence of dissolved organic matter (DOM) enhances the solubility of metal ions in soil–water system by forming the soluble DOM-metal complex [16, 17]. The higher content of dissolved organic matter in the soils rich in OM implies in greater concentrations of aqueous DOM-metal complexes and then in less the binding energies. Phyllosilicates and metal oxides are the predominant soil components on which metal ions are adsorbed. Metal sorption on soil hydrous oxides is a type of inner-sphere complexation [39], while the mechanisms of metal sorption on phyllosilicates is dominated by cation exchange, outer-sphere complexation and inner-sphere complexation or even precipitation [40, 41]. Therefore, metal ions adsorbed on phyllosilicates and metal oxides were not easily dissociated under the weaker applied electrical field strength. Meanwhile, there was a positive relationship between the measured binding energy (Cd or Zn) at different ratios and the content of phyllosilicates + metal oxides, i.e. the binding energy increased with the increase of the content of phyllosilicates + metal oxides. Not only soil type but also the ratio of Cd^2+^ to Zn^2+^ affects the partition of metal ions on the interface of soil–water. In addition, the binding energies of Cd^2+^ and Zn^2+^ all decreased with increasing the proportion of metal ions in soil particles, which is consistent with what Tiller et al. observed [38].

### Mean Gibbs free adsorption energy

**Fig. 5:**
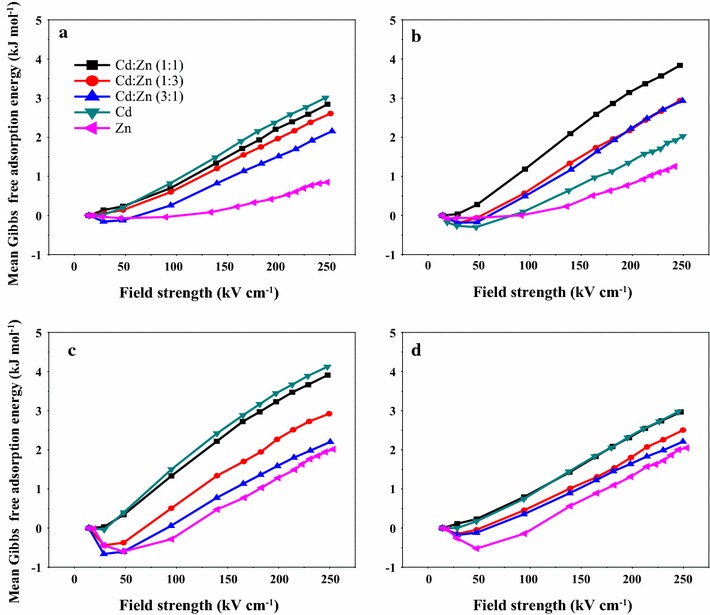
Mean free adsorption energies as function of field strength for suspension (10 g L^−1^) of clay fractions (< 2 μm) of paddy soil (Inceptisols) (LY) (**a**), boggy soil (Histosols) (JH) (**b**), yellow brown soil (Haplic Luvisols) (NJ) (**c**) and yellow drab soil (Ustalfs) (XY) (**d**) saturated with different ratios of Cd^2+^–Zn^2+^ in deionized water

The adsorption energies of metal ions on soil particles as a function of applied field strength were evaluated according to the calculation method proposed by previous studies. An indication of electrically adsorbing strength was used to identify the detachment tendency of ions from soil particles by external force provided by the applied electrical field. The greater the adsorption energy is, the weaker is the electrically adsorbing strength of metal ions on the surfaces of soil particles. As Fig. 5 shows, the adsorption energies of ions on soil particles increased slowly with the increase of field strength and decreased (referred to as a negative Wien effect) at E < 50 kV cm^−1^, while the growth of adsorption energies was rapid at high field strength (E > 100 kV cm^−1^). However, the adsorption energies decreased on the order of 1:1 > 1:3 > 3:1 of Cd^2+^:Zn^2+^ ratio in different soil particles with an increase of the field strength above 100 kV cm^−1^. At a Cd^2+^:Zn^2+^ ratio of 1:1, the adsorbing strength of electrically bound ions to soil particles was weak, thus leading to detachment of more ions from soil particles under application of electrical field. In addition, more ions were easier to desorb in the combined system with 75% Zn^2+^ and 25% Cd^2+^, compared with the system with 25% Zn^2+^ and 75% Cd^2+^, at the same field strength. This suggests that the binding of Cd^2+^ with soil particles is tighter than Zn^2+^, which is consistent with the order of electronegativity of Cd^2+^ (1.7) and Zn^2+^ (1.6) [42, 43]. As McBride [44] predicted, the bonding preference of trace metal chemisorption was Cd^2+^ > Zn^2+^ in accordance with the electronegativity of trace metal. Also, Moreira and Alleoni [45] proposed that electrostatic adsorption is the main mechanism of Cd^2+^ and Zn^2+^ retention in soils. Consequently, the binding strength for Cd^2+^ adsorbed on soil particles is stronger than that for Zn^2+^ in view of the electronegativities of Cd and Zn. In addition, the Misono softness parameter calculated from ionic radius and ionization potential is also used to predict the tendency of metals to form complexes. The ability to form strong complexes for Cd^2+^ is stronger than for Zn^2+^, depending on the parameter [20].

In addition, the adsorption energies of ions in yellow brown soil and boggy soil were 2.2–3.9 kJ mol^−1^ (Table 4), which are larger than those in paddy soil and yellow drab soil (2.2–3.0 kJ mol^−1^) (Table 4) at 200 kV cm^−1^ in the combined system. Also, the results show that the adsorption energies of ions in paddy soil and yellow drab soil are lower in most of field strengths above 100 kV cm^−1^. This indicated that the ions adsorbed on the surfaces of yellow brown soil and boggy soil particles are easily dissociated in an applied electrical field, and paddy soil and yellow drab soil have stronger binding force for the two ion species. The mineralogy of soils were determined and shown in Table 2 in the manuscript. The dominant minerals of yellow brown soil and boggy soil were mainly montmorillonite–vermiculite group with have more charges and larger expansibility, while the main minerals of paddy soil and yellow drab soil were kaolinite group or hydromica group, with less charges or weaker expansibility. As Fig. 1 shown, the boggy soil and yellow brown soil have more negative charges and cation exchange capacity. Consequently, yellow brown soil and boggy soil had strong binding capacity for Cd^2+^ and Zn^2+^, while paddy soil and yellow drab soil had weak affinity for Cd^2+^ and Zn^2+^, resulting in high environmental risk.

**Table 4:** Mean free adsorption energies of ions adsorbed in soil colloid particles with four soil types at field strength of 100, 150, 200, 250 kV cm^−1^

Soil type	Ratio of Cd^2+^/Zn^2+^	100	150	200	250
		kV cm^−1^			
Boggy soil	1:1	1.18	2.09	3.14	3.84
	1:3	0.57	1.33	2.17	2.93
	3:1	0.49	1.17	2.22	2.93
	Cd^2+^	0.09	0.64	1.35	2.03
	Zn^2+^	0.00	0.23	0.77	1.25
Paddy soil	1:1	0.70	1.34	2.20	2.84
	1:3	0.60	1.20	1.96	2.61
	3:1	0.26	0.82	1.51	2.15
	Cd^2+^	0.82	1.48	2.37	3.01
	Zn^2+^	− 0.04	0.08	0.53	0.85
Yellow brown soil	1:1	1.33	2.22	3.23	3.91
	1:3	0.51	1.34	2.27	2.92
	3:1	0.06	0.78	1.59	2.20
	Cd^2+^	1.50	2.42	3.45	4.12
	Zn^2+^	− 0.28	0.48	1.28	2.02
Yellow drab soil	1:1	0.79	1.43	2.31	2.97
	1:3	0.46	1.01	1.80	2.50
	3:1	0.35	0.89	1.64	2.21
	Cd^2+^	0.74	1.44	2.31	2.97
	Zn^2+^	− 0.14	0.56	1.32	2.05

## Conclusion

Soil heavy metal contamination is not only single-metal ion contamination but also a binary pollution. In this study, the suspension Wien effect was used to investigate the interactions between two species of heavy metal ions and four types of soil clay fraction particles in the Cd^2+^–Zn^2+^ binary systems at various ratios of Cd^2+^ to Zn^2+^. The binding energy and adsorption energy spectra of Cd^2+^ and Zn^2+^ for the soil clay fraction particles were calculated based on the EC-E curves. Compared to the binding energies of Cd^2+^ and Zn^2+^ in the mono-metal system, more Zn^2+^ was electrostatically adsorbed on the clay fraction particles of the four studied soils than Cd^2+^. The binding energy results in the competitive system suggested that Cd^2+^ is inclined to combine with the clay fraction particles of boggy soil and Zn^2+^ tend to combine with clay fraction particles of yellow brown soil. For paddy soil, Zn^2+^ and Cd^2+^ are prone to partition into soil solution, resulting in the increase of the mobility and bioavailability of metal ions. Although soil clay fraction particles have a strong attraction to certain ions, the binding force between ions and soil particles may be weak. The adsorption energies of ions in boggy soil and yellow brown soil were higher, indicating weaker electrically adsorbing strength between ions and soil particles. Consequently, mobility and bioavailability of Cd^2+^ and Zn^2+^ are poor in boggy soil and yellow brown soil, respectively, while the potential mobility and bioavailability of Cd^2+^ and Zn^2+^ are high with the external force provided by the applied electrical field. In the combined system there was antagonism between Cd^2+^ and Zn^2+^ resulting in a weaker binding force between ions and soil particles at a 1:1 ratio of Cd^2+^–Zn^2+^. Furthermore, the affinity of ions to soil particles decreased with the increase of the proportion of ions (Cd^2+^ or Zn^2+^) in the Cd^2+^–Zn^2+^ binary samples.
